# Children’s Health: Breastfeeding: Nature’s MRE

**DOI:** 10.1289/ehp.114-a25a

**Published:** 2006-01

**Authors:** Julia R. Barrett

Low breastfeeding rates and inadequate emergency planning left many infants dehydrated and hungry in the wake of Hurricane Katrina. Health and educational organizations responded rapidly with breastfeeding information and assistance. Through direct contact with mothers and emergency responders, the groups strove to implement long-standing international guidelines for feeding infants in emergencies.

Breastfeeding provides optimal nutrition, protection against infection, and a safe, reliable food source for babies—attributes that are critical in emergencies. International health organizations including the World Health Organization (WHO) and the United Nations Children’s Fund (UNICEF) promote breastfeeding as the best way to feed infants in a crisis. Although formula is an adequate substitute when a child does not receive breast milk, it must be available with a supply of clean water and containers, and instructions for feed preparation must also be available. Yet potable water, formula itself, and even mixing containers may be impossible to acquire in an emergency.

The WHO and UNICEF have long had guidelines that strongly favor breastfeeding in crises. Current guidelines stem in part from the March 1999 Kosovo crisis in which war forced thousands of Kosovar Albanians into refugee camps. Andrew Seal, a lecturer in international nutrition at the London-based Institute of Child Health and coauthor of a 1999 report based on the Kosovo experience, says, “I think the guidelines are quite good, but it’s like any other specific technical sector—it depends on having people within the organization who have the interest and awareness to champion that particular cause when there are one thousand and one other things to be thinking about.”

Breastfeeding should begin at birth, but a full milk supply can be established even several days after birth. If a nonbreastfed infant is less than six months old, a mother may be able to relactate; beyond that, it is sometimes possible to induce lactation for a partial milk supply. Health organizations dispute the common beliefs that stress “dries up” a mother’s milk and that malnourished mothers cannot produce milk, but emphasize that optimal breastfeeding requires a supportive environment.

Guidelines issued by the American Academy of Pediatrics in 2005 emphasize that children younger than six months old require no other food or fluids beyond breast milk and recommend that breastfeeding continue after solid foods are introduced for at least the first year of life or longer if mother and child wish to continue. The WHO and UNICEF recommend breastfeeding for at least two years.

One significant problem in the Gulf Coast crisis was a lack of breastfeeding knowledge in the affected population. “We sent . . . board-certified lactation consultants into the shelters to start working directly with the mothers who wanted our help,” says Katy Lebbing, herself an international board-certified lactation consultant with La Leche League International, an organization that supports and promotes breastfeeding. But few women were already breastfeeding. “Not only did we have to help people with breastfeeding, but we also had to educate people about breastfeeding,” she says.

Getting breastfeeding support and information to people in crisis is problematic, though. Says Seal, “We need integrated interventions that acknowledge the reality of a mother’s established feeding decisions.”

Indeed, one reality is that breastfeeding rates are extremely low in many areas, including Louisiana and Mississippi, which have some of the lowest breastfeeding rates in the nation, according to the Centers for Disease Control and Prevention. Nevertheless, Lebbing hopes that breastfeeding promotion efforts after Katrina planted a seed. “Natural disasters and other types of disasters happen,” she says. “The best choice is to breastfeed because you don’t have to worry about your baby’s milk supply.”

## Figures and Tables

**Figure:**
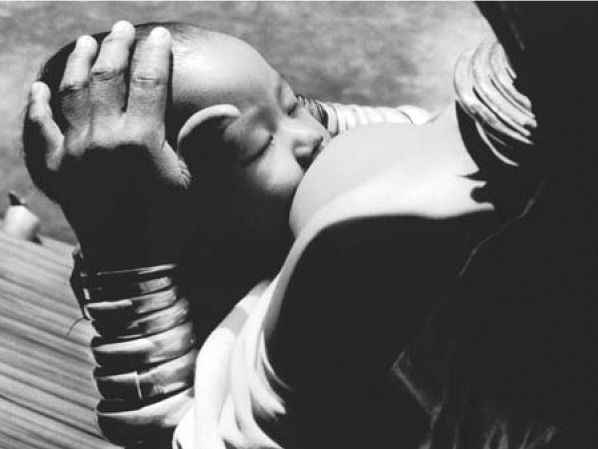
Comfort food. Breastfeeding, as in this refugee camp in Thailand’s Mae Hong Son Province, is best for infants in emergency situations.

